# Effects of flaxseed oil supplementation on metaphase II oocyte rates in IVF cycles with decreased ovarian reserve: a randomized controlled trial

**DOI:** 10.3389/fendo.2024.1280760

**Published:** 2024-02-26

**Authors:** Qi Chu, Yue-xin Yu, Jing-zi Zhang, Yi-tong Zhang, Jia-ping Yu

**Affiliations:** Department of Reproductive Medicine, General Hospital of Northern Theater Command, Shenyang, Liaoning, China

**Keywords:** decreased ovarian reserve, flaxseed oil, metaphase II oocyte rates, omega-3 fatty acids, human follicle-stimulating hormone

## Abstract

**Background:**

This study was designed to explore the effects of flaxseed oil on the metaphase II (MII) oocyte rates in women with decreased ovarian reserve (DOR).

**Methods:**

The women with DOR were divided into a study group (n = 108, flaxseed oil treatment) and a control group (n = 110, no treatment). All patients were treated with assisted reproductive technology (ART). Subsequently, the ART stimulation cycle parameters, embryo transfer (ET) results, and clinical reproductive outcomes were recorded. The influencing factors affecting the MII oocyte rate were analyzed using univariate analysis and multivariate analysis.

**Results:**

Flaxseed oil reduced the recombinant human follicle-stimulating hormone (r-hFSH) dosage and stimulation time and increased the peak estradiol (E2) concentration in DOR women during ART treatment. The MII oocyte rate, fertilization rate, cleavage rate, high-quality embryo rate, and blastocyst formation rate were increased after flaxseed oil intervention. The embryo implantation rate of the study group was higher than that of the control group (*p* = 0.05). Additionally, the female age [odds ratio (OR): 0.609, 95% confidence interval (CI): 0.52–0.72, *p* < 0.01] was the hindering factor of MII oocyte rate, while anti-Müllerian hormone (AMH; OR: 100, 95% CI: 20.31–495, *p* < 0.01), peak E2 concentration (OR: 1.00, 95% CI: 1.00–1.00, *p* = 0.01), and the intake of flaxseed oil (OR: 2.51, 95% CI: 1.06–5.93, *p* = 0.04) were the promoting factors for MII oocyte rate.

**Conclusion:**

Flaxseed oil improved ovarian response and the quality of oocytes and embryos, thereby increasing the fertilization rate and high-quality embryo rate in DOR patients. The use of flaxseed oil was positively correlated with MII oocyte rate in women with DOR.

**Clinical trial number:**

https://www.chictr.org.cn/, identifier ChiCTR2300073785

## Introduction

The ovarian reserve reflects the sum of follicles in the ovary. With the increase in women’s age, their fertility decreases, as well as their ovarian reserve function and the number and quality of oocytes (1, 2). Compared with women of the same age, women with decreased ovarian reserve (DOR) have lower fecundity and responsiveness to exogenous ovarian hormone stimulation, resulting in less oocyte retrieval, poorer embryo quality, and lower implantation rate and pregnancy rate (2, 3). Additionally, DOR patients may be also characterized by perimenopausal symptoms, such as irregular menstruation, sleep disorder, and mood fluctuations (3). Currently, DOR is one of the most important therapeutic challenges in assisted reproduction (4, 5).

The decline in fertility caused by DOR has attracted attention. A woman with DOR may suffer from ovarian hypo-response, increased use of ovulation stimulants, and a high miscarriage rate after receiving assisted reproductive technology (ART) (6). These difficulties increase the psychological burden on patients and reduce the possibility of achieving parenthood. A previous study reported that DOR affected the quantity and number of metaphase II (MII) oocytes during ART treatment (7). Therefore, it is necessary to explore the treatment methods to improve the pregnancy outcomes of DOR patients.

Flax is a traditional plant, and flaxseed oil is extracted from the seeds of plant flax. Flaxseed oil is rich in omega-3 fatty acids required for human health, of which α-linolenic acid (ALA) is the most abundant (8, 9). As confirmed by previous studies, flaxseed oil plays a role in the human reproductive system, such as promoting follicular development, improving oocyte quality, and even improving oocyte fertilization rate (10, 11). Animal researchers have reported that flaxseed oil can be used as a supplement to improve reproductive processes (12, 13). However, the effects of flaxseed oil on the MII oocytes in DOR patients have not been reported yet. Therefore, this study focused on the investigation of the influence of flaxseed oil on the MII oocyte rates of women with DOR in *in vitro* fertilization (IVF)-assisted pregnancy.

## Materials and methods

### Study design and randomization

**Figure 1:**
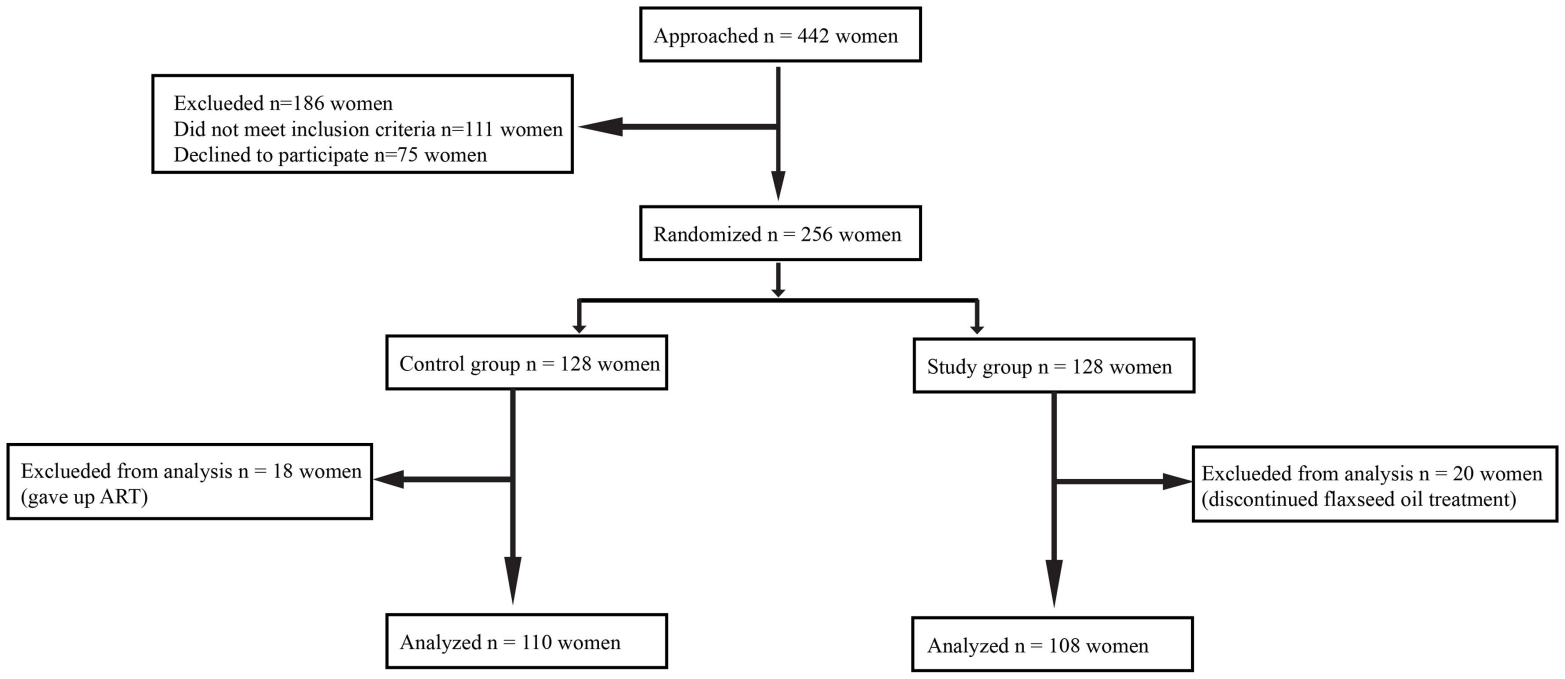
The chart of experimental flow.

This was a prospective, randomized controlled study conducted at the Reproductive Medical Center of General Hospital of Northern Theater Command from April 1, 2021, and June 30, 2022. The experimental flow in this study is presented in **Figure 1**.

All participants were randomized 1:1 to either the study group or the control group. The randomization was performed over the period of 14 months (between April 1, 2021, and June 30, 2022) using computer-generated randomization codes. The study participants and the investigators were not blinded to the patient grouping. Additionally, this study was approved by the Ethics Committee of the General Hospital of Northern Theater Command (Y(2021)-089). All procedures were in accordance with the ethical guidelines and the Declaration of Helsinki. Informed consent was obtained from all patients.

### Participants

Inclusion criteria were shown as follows: 1) women aged ≤40 years; 2) anti-Müllerian hormone (AMH) <1.2 ng/ml; 3) antral follicle count (AFC) <7; 4) follicle-stimulating hormone (FSH) greater than 10 and less than 25.

Exclusion criteria were as follows: 1) women aged >40 years; 2) suffering from gynecological diseases of the ovaries (ovarian tumors, ovarian cysts, and endometriosis); 3) having taken ovarian stimulation drugs or received controlled hyperovulation therapy within 6 months; 4) with history of ovarian surgery; 5) with endocrine or autoimmune disease (e.g., diabetes, thyroid disease, and polycystic ovary syndrome); 6) having taken drugs affecting the metabolism of macro- and micronutrients within 3 months including hypoglycemic and lipid-lowering drugs; 7) receiving prior antioxidant treatment or known allergy to flaxseed oil in the past 1 year.

### Treatment protocols

The patients in the study group were given oral flaxseed oil (ALA 500 mg/pill, Kings Healthbay, Walnut, CA, USA) twice a day for 30 days before receiving ART treatment. During ART treatment, oral drug doses were maintained until a clinical pregnancy was established. The subjects in the control group received ART before and after IVF or intracytoplasmic sperm injection (ICSI), without any additional treatment.

### Ovarian stimulation

On the second or third day of menstruation, recombinant human follicle-stimulating hormone (r-hFSH; Jinsai, China) was given at an initiative dose ranging from 150 to 225 U. The late dose of r-hFSH was adjusted according to the size and counts of the follicles and hormone levels. When the diameter of dominant follicles was ≥12–14 mm, gonadotropin-releasing hormone (GnRH) antagonist (Cetrorelix, 250 μg/day; Merck Serono, Darmstadt, Germany) was administered until the follicles were mature. After that, recombinant human chorionic gonadotrophin (hCG) (Ovidrel, 250 μg; Merck Serono S.p.A, Rome, Italy) was used to trigger ovulation. Thirty-six hours later, ovulation was triggered, and oocytes were extracted (14).

### Embryo culture

Oocytes were fertilized by conventional IVF or ICSI depending on sperm quality (15, 16). Embryos were evaluated 16–18 hours after IVF or ICSI fertilization, and zygotes normally fertilized were cultured in an embryo incubator for 3–5 days (17).

### Embryo transfer

The embryos were graded according to the consensus of the laboratory group of the Reproductive Medicine Branch of the Chinese Medical Association and the Istanbul consensus (18). All subjects selected the best morphologically graded embryos for fresh or frozen embryo transfer (ET) on the third day (19, 20). If the subjects were eligible for fresh transplantation, 40 mg of progesterone was injected intramuscularly from the day of oocyte extraction, 200-mg oral progesterone capsules were given per day, and luteal support was given until 14 days after ET (17). For subjects with a positive pregnancy, luteal support was provided until 8 weeks of gestation.

Those who failed to undergo fresh transfer were subjected to a frozen ET cycle at a later stage. Patients with a regular menstrual cycle and normal ovulation underwent the natural cycle program, and their ovulation status was tracked using transvaginal ultrasound. Meanwhile, blood luteinizing hormone (LH), estradiol (E2), and progesterone were monitored. The patients were given progesterone 20 mg/day of intramuscular injection on ovulation day and a progesterone capsule 200 mg/day orally to transform the endometrium. ET was performed 3 days after ovulation with luteal support until 8 weeks of gestation.

As for menstrual irregularities, artificial cycles were applied in the endometrium of subjects with ovulatory disorders. From the fifth day of the menstrual cycle, 2-mg fenmoton white tablets (E2 tablets) were orally given to patients twice a day, and their endometrial thickness was monitored by transvaginal ultrasonography. When the endometrial thickness exceeded 8 mm, luteal support (ketone injection 40 mg combined with oral progesterone capsules 200 mg/day) was given, and ET was performed 5 days later. Hormone therapy was discontinued if the pregnancy test result was negative. A positive pregnancy test resulted in luteal support up to 11 weeks of gestation and tapering after 10 weeks.

### Outcome measures

The primary outcome measure was the MII oocyte rate for one stimulation cycle. The MII oocyte rate was defined as the ratio of the total number of MII oocytes to the total number of oocytes extracted.

The secondary outcomes included stimulation cycle parameters, embryology-related parameters, and clinical reproductive outcome parameters. Specifically, stimulation cycle parameters consisted of the total dose of r-hFSH, duration of stimulation, peak E2 concentration, LH concentration on the day of the hCG trigger, endometrial thickness on the day of the hCG trigger, duration between the day of the hCG trigger and oocyte collection, and canceled cycles. Embryology-related parameters were composed of the percentage of ICSI, fertilization rate, cleavage rate, day 3 high-quality embryo rate, and blastocyst development rate. Clinical reproductive outcome parameters were composed of the number of fresh or frozen ET cycles, clinical pregnancy rate per fresh ET, implantation rate, cumulative clinical pregnancy rate, multiple pregnancy, and spontaneous miscarriage.

The fertilization rate was defined as the number of fertilized oocytes developed by the number of MII oocytes. Cleavage rate was defined as the number of fertilized oocytes divided into embryos divided by the total number of fertilized oocytes. Day 3 high-quality embryo rate was defined as the number of good-quality embryos divided by the number of all embryos. Clinical pregnancy was defined as the presence of an intrauterine gestational sac observed on ultrasound after ET. The cumulative clinical pregnancy rate was defined as the number of clinical pregnancies resulting from the index ART cycle following fresh or frozen ET divided by the number of all women who received treatment. Multiple pregnancy was defined as the simultaneous presence of two or more gestational sacs in the uterine cavity after a single transplant. Spontaneous miscarriage was defined as a loss of clinical pregnancy before 24 weeks of gestation.

### Statistical analysis

Continuous variables were expressed as mean ± standard deviation (SD) (normal distribution) or median (quartile) (skewed distribution). Categorical variables were expressed in frequency or as a percentage. For normally distributed variables, a t-test was used to compare differences between groups. For non-normally distributed variables, differences between groups were compared using the Mann–Whitney U test. The chi-squared test was employed for comparisons of categorical variables. Univariate and multivariate logistic regression analyses were performed, and MII oocyte rates ≥50% were defined as a positive event. Univariate logistic regression analyses were performed to assess potential predictors associated with MII oocyte rates. The influence factors with statistical differences (*p* < 0.05) in univariate analyses were subject to multivariate logistic regression analyses. All analyses were performed using SPSS 22.0 software. *p* < 0.05 indicated significant differences.

## Results

### Overall conditions and baseline characteristics of patients

A total of 442 women were recruited. Subsequently, 186 women were excluded because they failed to meet the inclusion criteria (n = 111) or declined to participate (n = 75). The remaining 256 women agreed to participate in the study, and they were randomly assigned to a study group (n = 128) or a control group (n = 128). Additionally, 38 subjects were excluded from the analysis for the following reasons: 18 women changed their minds and gave up ART, and 20 women discontinued flaxseed oil treatment due to adherence problems. Finally, 108 women were retained in the study group and 110 in the control group. Baseline characteristics of the two groups were comparable in terms of age, body mass index (BMI), duration of infertility, type of infertility, other causes of infertility, and ovarian reserve (AMH, AFC, and day 3 FSH). The baseline characteristics of the two groups are displayed in **Table 1**.

**Table 1:** Baseline characteristics of two groups.

Variables	Study group (n = 108)	Control group (n = 110)	*p*
Female age (years)	35 ± 2.54	34.4 ± 3.02	0.15
BMI (kg/m^2^)	23.9 ± 4.24	23.5 ± 3.47	0.41
Infertility duration (years)	3.97 ± 3.23	4.17 ± 3.46	0.66
Primary infertility, n (%)	45 (41.7%)	51 (49.1%)	0.48
Diagnosis of infertility in addition to DOR, n (%)			
Male factor	22 (20.4%)	26 (23.6%)	0.56
Tubal factor	38 (35.2%)	45 (41.0%)	0.38
Adverse pregnancy and birth history, n (%)	15/108	14/110 (12.7%)	0.80
Ovarian reserve markers			
AMH (ng/ml)	0.66 ± 0.31	0.60 ± 0.32	0.42
AFC (number)	3 (2,4)	3 (2,4)	0.08
Day 3 FSH (IU/ml)	16.2 ± 2.50	16.2 ± 2.56	0.96

Data are expressed as mean ± SD, n (%), or median (IQR).

BMI, body mass index; DOR, decreased ovarian reserve; AMH, anti-Müllerian hormone; AFC, antral follicle count; FSH, follicle-stimulating hormone.

### Comparison of ART stimulation cycle parameters and embryological results between two groups

ART stimulation cycle parameters and embryological results are summarized in **Table 2**. The total dose and the duration of stimulation of r-hFSH in the study group were lower than those in the control group (*p* = 0.04 and 0.03). The peak E2 concentration in the study group was significantly higher than that in the control group (*p* = 0.01). There was no significant difference between the two groups in terms of endometrial thickness on the day of the hCG trigger (*p* = 0.11) and the interval between the day of the hCG trigger and oocyte retrieval (*p* = 0.19).

**Table 2:** ART cycle stimulation parameters and embryology outcomes of two groups.

Variables	Study group (n = 108)	Control group (n = 110)	*p*
Cycle stimulation			
Total dose of r-hFSH (IU)	2,111 ± 788	2,233 ± 900	0.04
Duration of stimulation (days)	9.34 ± 2.96	9.78 ± 3.04	0.03
Peak E2 concentration (pmol/L)	1,212 ± 792	1,026 ± 1,114	0.01
Endometrial thickness on the day of hCG trigger (mm)	0.98 ± 0.25	1.02 ± 0.21	0.11
Duration between the day of hCG trigger and oocyte collection (hours)	36.2 ± 0.65	36.1 ± 0.66	0.19
Embryology outcomes			
MII oocyte rate (%)	287/339 (84.6%)	292/370 (78.9%)	0.05
ICSI (%)	68/108 (63.0%)	59/110 (53.6%)	0.16
Fertilization rate (%)	221/287 (76.9%)	207/292 (70.9%)	0.02
Cleavage rate (%)	141/221 (63.8%)	107/207 (51.7%)	0.01
Day 3 high-quality embryo rate (%)	155/287 (54.0%)	133/292 (45.6%)	0.04
Blastocyst development rate (%)	67/117 (57.3%)	50/114 (43.9%)	0.04

Data are expressed as mean ± SD or n (%).

r-hFSH, recombinant human follicle-stimulating hormone; E2, estradiol; hCG, human chorionic gonadotrophin; MII, metaphase II; ICSI, intracytoplasmic sperm injection.

There was no statistical difference in fertilization method between the two groups, and the proportions of ICSI in the two groups were 64.0% and 53.6%, respectively (*p* = 0.16). Although not statistically significant, the MII oocyte rate was greater in the study group (84.6%) compared to the control group (78.9%). The fertilization rate (76.9% vs. 70.9%) and cleavage rate (63.8% vs. 51.7%) in the study group were higher than those in the control group, and there was a statistical difference. In addition, the day 3 high-quality embryo rate and blastocyst formation rate in the study group were higher than those in the control group. Furthermore, flaxseed oil intervention not only improved oocyte quality and embryo quality but also increased MII oocyte rate, fertilization rate, cleavage rate, high-quality embryo rate, and blastocyst formation rate.

### Comparison of clinical reproductive outcomes between two groups

The clinical reproductive outcomes of the two groups of subjects are summarized in **Table 3**. The cycle cancellation rate was not statistically different between the two groups (*p* = 0.80). In the study group, 32 subjects received fresh transfer, and 70 received frozen ET. In the control group, 26 subjects received fresh transfer, and 67 subjects received frozen ET. The fresh transfer cycle of the two groups was less than the frozen ET cycle, and there was no statistical difference. However, the embryo implantation rate of the study group (51.9%) was higher than that of the control group (45.5%, *p* = 0.04). Although the cumulative clinical pregnancy rate of the study group was higher than that of the control group, there was no significant difference (*p* = 0.81). In addition, there was no significant difference in the rates of multiple pregnancy and early miscarriage between the two groups.

**Table 3:** Clinical reproductive outcomes of two groups.

Variables	Study group (n = 108)	Control group (n = 110)	*p*
Canceled cycles (%)	6/108 (5.56)	7/110 (6.36)	0.80
Number of fresh ET cycles (%)	32/108 (29.6)	26/110 (23.6)	0.32
Number of frozen ET cycles (%)	70/108 (64.8)	67/110 (60.9)	0.55
Implantation rate (%)	56/108 (51.9)	50/110 (45.5)	0.04
Cumulative clinical pregnancy rate (%)	41/108 (38.0)	40/110 (36.4)	0.81
Multiple pregnancy (%)	11/108 (10.2)	13/110 (11.8)	0.70
Spontaneous miscarriage (%)	12/108 (11.1)	14/110 (12.7)	0.71

ET, embryo transfer.

### Multivariate regression analysis for factors influencing MII oocyte rates

An analysis was performed on the influencing factors for MII oocyte rates. The results of the univariate analysis showed that the influencing factors for MII oocyte rates included female age, AMH, AFC, peak E2 concentration, and use of flaxseed oil (**Table 4**). Female age was significantly negatively correlated with MII oocyte rate (*p* = 0.01), while AMH (*p* = 0.05), AFC (*p* = 0.03), peak E2 concentration (*p* = 0.01), and flaxseed oil intake (*p* = 0.05) were positively correlated with MII oocyte rate.

**Table 4:** Univariate analysis of MII oocyte rate.

Variables	OR (95% CI)	*p*
Female age (years)	1.81 (1.15–2.85)	0.01
BMI (kg/m^2^)	0.97 (0.93–1.00)	0.56
Infertility duration (years)	1.00 (0.97–1.04)	0.96
Types of infertility	0.77 (0.58–1.02)	0.06
Infertility factor	0.93 (0.66–1.30)	0.66
AMH (ng/ml)	1.59 (1.00–2.51)	0.05
AFC (number)	1.12 (1.01–1.23)	0.03
Day 3 FSH (IU/ml)	1.02 (0.98–1.07)	0.38
Total dose of r-hFSH (IU)	1.00 (1.00–1.00)	0.56
Duration of stimulation (days)	1.00 (0.97–1.04)	0.91
Peak E2 concentration (pmol/L)	1.00 (1.00–1.00)	0.01
Endometrial thickness on the day of hCG trigger (mm)	0.88 (0.54–1.43)	0.62
Duration between the day of hCG trigger and oocyte collection (hour)	0.96 (0.89–1.04)	0.32
Flaxseed oil intake	1.33 (1.01–1.75)	0.05

OR, odds ratio; CI, confidence interval; BMI, body mass index; AMH, anti-Müllerian hormone; AFC, antral follicle count; FSH, follicle-stimulating hormone; r-hFSH, recombinant human follicle-stimulating hormone; E2, estradiol; hCG, human chorionic gonadotrophin.

We further analyzed the association between MII oocyte rate and these influencing factors using multiple-factor analysis (**Table 5**). Briefly, the female age [odds ratio (OR): 0.61, 95% confidence interval (CI): 0.52–0.72, *p* < 0.01] was the hindering factor, while AMH (OR: 100, 95% CI: 20.3–495, *p* < 0.01), peak E2 concentration (OR: 1.00, 95% CI: 1.00–1.00, *p* = 0.01), and the intake of flaxseed oil (OR: 2.51, 95% CI: 1.06–5.93, *p* = 0.04) were the promoting factors for MII oocyte rate. These results indicated that the increase in female age was related to the decrease in MII oocyte rate. Additionally, high AMH and E2 levels and flaxseed oil treatment contributed to the high MII oocyte rate.

**Table 5:** Multivariate analysis of MII oocyte rate.

	Multivariate analysis	
Variables	OR (95% CI)	*p*
Female age (years)	0.61 (0.52–0.72)	<0.01
AMH (ng/ml)	100 (20.3–495)	<0.01
AFC (number)	1.25 (0.94–1.67)	0.13
Peak E2 concentration (pmol/L)	1.00 (1.00–1.00)	0.01
Flaxseed oil intake	2.51 (1.06–5.93)	0.04

OR, odds ratio; CI, confidence interval; AMH, anti-Müllerian hormone; AFC, antral follicle count.

## Discussion

DOR is characterized by a decrease in the quality and quantity of oocytes (1, 2). It has seriously affected the fertility of women and is progressively worse with the delay of childbearing age (2). The prevalence of DOR is approximately 10% among infertile women (21). Previous studies have shown that flaxseed oil can improve fertility (10, 11). In this study, we demonstrated the potential benefits of flaxseed oil treatment in improving oocyte quality and ovarian response in women with DOR. Our results showed that the flaxseed oil intervention could improve ovarian response, reduce r-hFSH dosage, shorten r-hFSH stimulation time, and increase E2 peak concentration. Furthermore, flaxseed oil intervention could improve oocyte quality and embryo quality and increase the rate of mature oocyte acquisition, fertilization rate, cleavage rate, high-quality embryo rate, and blastocyst formation rate. Additionally, the embryo implantation rate after flaxseed oil treatment was higher than that of the control group. The cumulative clinical pregnancy rate was higher after flaxseed oil treatment, but there was no significant difference, probably due to insufficient sample size. In conclusion, flaxseed oil administration enhanced ovarian response to stimulation and improved oocyte and embryo quality. The results of this study are generally consistent with those of some previous studies.

The intake of linseed oil can increase the levels of ALA and eicosapentaenoic acid in the body (22). These two substances are very important for germ cell development. Specifically, they play a role in maintaining the structure and function of cell membranes, enhancing immune function, promoting growth and development, and regulating lipid metabolism and related gene expression (23, 24). A study of 235 women who underwent IVF/ICSI revealed that a high intake of ALA was associated with a higher baseline E2 level. They also showed positive associations between ALA intake and embryo morphology (25). Another study pointed out that total oocyte volume and MII oocyte volume were positively correlated with ALA intake, and a higher intake of linseed oil increased the oocyte fertilization rate (11). The beneficial effect of flaxseed oil on the fertility of women may be related to omega-3 fatty acids. A randomized controlled study of 110 participants proposed that dietary intervention with omega-3 fatty acids could significantly improve embryo development through morphodynamic markers improving embryo quality (26). In a randomized controlled trial of 27 participants, dietary supplementation with omega-3 fatty acids reduced serum FSH levels in normal-weight women (27). This is consistent with the direction of data in mice that higher dietary omega-3 fatty acids can increase reproductive lifespan (27, 28). In addition, it was reported that the combination of flaxseed oil and vitamin E increased semen quality and sperm motility, prevented sperm lipid peroxidation, and increased blastocyst rate (29). Although our study did not pay attention to the effect of flaxseed oil on sperm, our results demonstrated the beneficial effects of flaxseed oil on reproduction and were not limited to a single gender.

In our study, flaxseed oil intervention increased the rate of mature oocyte acquisition and follicle formation. This may be related to the effects of omega-3 fatty acids. Evans et al. and Mossa et al. suggested that dietary omega-3 fatty acids increased ovarian follicle production, which may have a positive effect on fertility performance (30, 31). Studies in cattle have shown that omega-3 fatty acids promote follicle growth in the ovaries, in addition to shortening the interval between first ovulation after delivery (32). The possible mechanism is that high omega-3 fatty acid levels increase arachidonic acid (AA) in the phospholipid of follicular granulosa cells. Under the stimulation of gonadotrophin, AA is released from the phospholipid and metabolized through the cyclooxygenase pathway to produce prostaglandins. Prostaglandin E2 stimulates ovarian hormone production and then increases follicles (33–35). It also may be associated with the antioxidant capacity of omega-3 fatty acids (36).

Omega-3 fatty acids may influence many factors related to the synthesis and metabolism of important reproductive hormones, such as steroid hormones, progesterone, and E2 (37, 38). It was found that the follicular fluid progesterone concentration of ewes fed with the omega-3 fatty acids diet was significantly higher than that of the control group (39). Diets rich in omega-3 fatty acids promote early embryonic development and progesterone secretion, suggesting that sex steroid metabolism may be affected by regulating omega-3 fatty acids intake (40). To be specific, inhibition of prostaglandin-endoperoxide synthase 2 (PTGS2) activity can promote cAMP-induced steroidogenesis in mouse Leydig tumor cells by increasing the expression of steroidogenic acute regulatory (STAR) protein, and omega-3 fatty acids are effective inhibitors of PTGS2 activity (41). In this study, the E2 peak level was associated with the MII oocyte rate, and flaxseed oil increased the peak E2 concentration in DOR women.

Our study showed that flaxseed oil intervention improved the quality of oocytes and embryos and increased fertilization rate, high-quality embryo rate, and blastocyst formation rate. Additionally, omega-3 fatty acids could increase fertilization rate and promote embryo development. Notably, high levels of omega-3 fatty acids improve membrane fluidity, reduce embryo fragmentation, and make blastocyst division more (42)symmetrical; all of these are associated with increased implantation, high live birth rates, and improved blastocyst development (25, 43). It has also been suggested that omega-3 fatty acids increase insulin-like growth factor-I (IGF-I) gene expression in granulosa cells (44, 45), thereby improving fertilization rates and embryonic development. IGF-1 is a key regulator of follicular differentiation and other reproductive functions (42, 46, 47). Our study did not analyze the outcomes of fresh or frozen ET with flaxseed oil. We suspected that flaxseed oil may have no effect on the method of transplantation (fresh or frozen). Previous studies have stated that omega-3 fatty acids affect oocyte development mainly through antioxidants, steroid metabolism, gene expression, and other mechanisms (48–50). Therefore, it is speculated that flaxseed oil may mainly function by targeting the effect of ovulation induction on the oocytes of subjects. However, some studies have stated that the pregnancy outcome of fresh and frozen ET cycles may be mainly related to the quality of transplanted embryos and the endometrial environment (51, 52). Perhaps, more clinical and basic studies are needed in the future to confirm the effects of linseed oil omega-3 fatty acids on the endometrial environment and embryo quality.

The main strength of this study is that the impact of flaxseed oil on MII oocyte rate in patients with DOR has not been reported yet. Our study population was focused on a specific population of women with DOR, applying the same clinical treatment protocol and laboratory testing methods and using a prospective design of an unbiased randomization process to eliminate bias.

There are several limitations in this study. First of all, the sample size in this study was small, so a larger sample size may be required to account for significant clinical differences. Second, the pregnancy outcomes related to live births were not tracked in this study. The pregnancy outcomes related to live births are the final outcome of IVF treatment, and a longer follow-up may be needed to evaluate patients. Furthermore, the effect of other lifestyle factors that may have put women at higher risk was not assessed in this study, and these factors need to be considered in subsequent studies. Finally, the optimal time and duration of flaxseed oil supplementation in this study were unclear. Considering the patient’s need for pregnancy assistance, lengthy intervention may not be accepted by patients, so the non-intervention time of 1 month was selected. In future research, the intervention time can be explored by further grouping. In other words, future research can explore the optimal intervention program for flaxseed oil by adjusting the intervention time and duration.

## Conclusion

Flaxseed oil improved ovarian response and the quality of oocytes and embryos, thereby increasing the fertilization rate and high-quality embryo rate in DOR patients. Furthermore, the use of flaxseed oil was positively correlated with MII oocyte rate in women with DOR.

## Data availability statement

The original contributions presented in the study are included in the article/supplementary material. Further inquiries can be directed to the corresponding author.

## Ethics statement

The studies involving humans were approved by General Hospital of Northern Theater Command (Y(2021)-089). The studies were conducted in accordance with the local legislation and institutional requirements. Written informed consent for participation in this study was provided by the participants’ legal guardians/next of kin.

## Author contributions

QC: Conceptualization, Investigation, Writing – review & editing. YY: Data curation, Formal analysis, Writing – original draft. JZ: Data curation, Formal analysis, Writing – original draft. YZ: Data curation, Formal analysis, Writing – original draft. JY: Conceptualization, Methodology, Writing – review & editing.
